# Evidence of local structural influence on the shape driven magnetic anisotropy in electronically excited Ni nanoparticles embedded in SiO_2_ matrix

**DOI:** 10.1038/s41598-017-18731-x

**Published:** 2018-01-18

**Authors:** Debalaya Sarker, Saswata Bhattacharya, H. Kumar, Pankaj Srivastava, Santanu Ghosh

**Affiliations:** 1 Department of Physics, Indian Institute of Technology Delhi, Hauz Khas, New Delhi, 110016 India; 2 Present Address: Department of Applied Physics, Amity University U.P., Sector 125 Noida, Noida, 201301 India

## Abstract

The reliance of modern electronic era on ultrafast data recording has made the search for novel tools to tune nano-scale magnetic-anisotropy (MA) never-ending. We demonstrate a strong correlation between the spin-spin interactions, local atomic structure and the MA of Ni nanoparticles (NPs) embedded inside SiO_2_ matrix under swift heavy ion (SHI) irradiation. In contrast to traditional understandings, MA in Ni NPs along with their aspect ratio, first increases upto 5 × 10^13^ ions/cm^2^ SHI fluence (5e13) and gets reduced at highest fluence. Using angle dependent Extented-Xray-Absorption-Fine-Structure (EXAFS) and *ab initio* molecular dynamics (MD) simulations, we show that the anisotropy induced in local atomic structure upon irradiation is dependent on atomic spin-spin interactions, which gets reduced at highest fluence. The chosen model cluster (Ni_38_) used in our MD simulations is duly validated by comparing the pair-correlation-function of the structure with the EXAFS-Fourier-Transform. The lattice temperatures for the films irradiated at different fluences, as calculated from thermal-spike-model, are used for the respective MD runs. We conclude that the enhanced disorder in both the local atomic environment and spin alignment destroys the MA at the highest fluence in SHI irradiated Ni NPs. The findings therefore provide rich conceptual insights for designing magnetic devices using SHI-induced phenomena.

## Introduction

Present day’s micro or nano electronics demand miniaturization of storage volume. One of the various activities in materials’ research to achieve this goal is perpendicular recording. This is undoubtedly superior to the commonly used in-plane storage technologies^1^. Thin films of ferromagnetic metals and alloys like Ni, Co, FeCo or FePt have attracted much attention in storage industry due to their large out-of-plane magneto crystalline anisotropy^2–4^. Alongside their thin films, these metals are studied extensively in their nanoparticles (NPs) form to achieve ultra high density magnetic storage^5,6^. Note that, NPs are not only beneficial in terms of gaining ultimate density limits of magnetic information storage, but also they don’t suffer from the interface/surface anisotropy effect, which is a major drawback in transition metal ultra-thin films^7^. Further, the tunability of the shape, size and orientation in NPs makes them more versatile for recording media as compared to the thin films. One of the smartest tricks is to elongate these NPs to tilt their easy axis of magnetization^8^. For this purpose, swift heavy ion (SHI) irradiation can be an efficient method^9,10^. Among different transition metals, Ni, being comparatively chemically stable, has been intensively investigated for its magnetic^11^, magneto-anisotropic^2^, magnetoresistive^12^, magnetostrictive^13^, and magneto-optical properties^14^, having applications in magnetic device physics. The particle size and/or size distribution, shape of NPs significantly affect various electronic properties controlling magnetic anisotropy (MA). Ni NPs with their strong spin-orbit coupling and a large magnetization, can promise a significant MA. Therefore it is important to address these factors and design experiments to tune MA before applying them in direct industrial parol. Note that, the phenomenon of shape anisotropy is associated with the zeroth spatial order of the magnetization field^15^ and produces the largest coercive forces^16^. Therefore, shape anisotropy is expected to align the magnetic easy axis of NPs with respect to the surface. Very small deviations from spherical shapes can significantly alter the coercivity and thus alterations in the coercive field is a direct measurement of MA.

The impressive development of accelerators in the last decade has brought forth swift heavy ion (SHI) irradiation as a promising tool for engineering materials down to nanoscale because of large electronic energy loss (S_*e*_). While interacting with NPs embedded within some insulating media (e.g. SiO_2_), high energy heavy ions can modify the crystallinity^17^ or MA^10^ and can elongate the NPs along^2,18,19^ or perpendicular^20^ to beam direction by transient temperature rise viz. thermal spike in the lattice subsystem^21–23^. In addition, local atomic disordering^24^, change in bond length, modification of surface^25^, etc are reported consequences of SHI-matter interaction. We have found that SHI mediated thermal spike induced high temperatures (≈4000–6000 K) modify various metal clusters viz. FeCo^23,24^ or Ni^25,26^ differently. The spin-spin interaction controls the local structural parameters in these ferromagnetic nanoclusters^26,27^. As a result of these local structural modifications, we have observed (i) triggering of the spin-flipping modulated exchange bias effect at high irradiation dose in FeCo NPs, (ii) reduction in Fe-Co nearest-neighbour distances (i.e. 1^*st*^ shell Fe-Co bond lengths)^27^. In Ni NPs, the spin-spin interaction affects the second nearest neighbour co-ordination^26^ and hence a local structural anisotropy is generated in post-irradiated NPs. This is why it is expected that the spin-driven local structural modifications will have a strong influence on MA of irradiated Ni NPs.

In this article, we have successfully tuned and optimized the MA in Ni NPs by varying SHI irradiation fluences. We have found that the MA first increases with irradiation fluence but then gets reduced as one goes beyond a certain higher fluence. Note that, the sudden reduction in MA beyond a particular intermediate fluence is not an usual observation for SHI irradiated ferromagnetic NPs viz. Co^10^, FePt^9^ etc. Therefore, a proper insight of this phenomenon is demonstrated with an in-depth experimental and theoretical analysis at the atomistic level. In the process of understanding, we have noted the impact of spin-spin interaction on the local atomic environment from a combined angle dependent EXAFS and DFT analysis, which is inter-winded with MA. We observed that the anisotropy introduced in the local atomic environment follows the same trend as the MA. The structural anisotropy is governed mainly from atomic spin-spin interactions. Above certain higher fluences, this spin-spin interaction and hence the structural anisotropy gets disrupted that in-turn causes the reduction in MA.

## Experimental and Theoretical Methods

Co-sputtering of Ni foils (99.9% purity, Sigma Aldrich) glued on SiO_2_ target (3 inch diameter, Sigma Aldrich) by 1.5 keV fast atom beam (FAB) of Ar was used for film preparation. Post deposition, thermal annealing in Ar-H_2_ environment at 750 °C for two hours was carried out. The composition and film thickness were measured by Rutherford backscattering spectrometry (RBS) using 1.7 MeV He^+^ ions at a scattering angle of 170°. A film thickness ≈440 nm and ≈10.5 ± 0.5% of Ni are obtained from RBS analysis in unirradiated film. The as deposited and annealed films were then subjected to different fluences (1 × 10^13^, 5 × 10^13^, 7.5 × 10^13^ and 1.0 × 10^14^ ions/cm^2^) of 100 MeV Au^+7^ beam: to be referred as 1e13, 5e13, 7.5e13 and 1e14 respectively from now onwards. Further details can be found in our earlier works^2,25,28–30^. To analyze irradiation induced MA, in-plane and out-of-plane SQUID measurements were performed [QD, MPMS XL-7] at 5 K. The underlying electronic and local atomic structures were then studied with x-ray absorption spectroscopy (both at near edge and far edges i.e. XANES and EXAFS) at XAFS beamline of Elettra synchrotron, Italy. The local atomic environments of Ni atoms in different films are probed from different directions by carrying out EXAFS measurements in 3 different incident angles of x-ray beam. At normal incidence (i.e. 10° EXAFS), the photoelectric vector E remains nearly parallel to the film surface and for the grazing incidence (80° EXAFS), E is almost parallel to the film normal. The usual EXAFS measurements, i.e. for 45° incident x-rays, are also carried out. Valence band of unirradiated (UI) and post-irradiated films were characterized by x-ray photoelectron spectroscopy (XPS) using Mg K*α* radiation.

Note that spin-orbit coupling monitors MA, which is directly related to the asymmetry of the atomic environment. Therefore, an insight of the observed MA effects is obtained here from both EXAFS and density functional theory (DFT) based calculations. We have used plane wave basis set as implemented in Vienna *ab initio* simulation package^31,32^ for our DFT study. Projector-augmented wave (PAW) pseudopotential is used in VASP for Ni atoms having 10 electrons in their valence shell. As role of insulating matrix is only to restrict the heat flow inside metal NPs, we have approximated our model system to be the metal clusters only. It’s been reported that 38 atoms Ni-cluster is one of the most stable structure of fcc Nickel^33,34^ and therefore, the global minimum structure of Ni_38_ cluster is chosen for our calculations. The PBE^35^ exchange correlation is used and it’s validated w.r.t HSE06^36^ hybrid functional wherever is needed.

The changes in the nanocomposite after SHI-matter interaction are quantitatively studied with thermal spike simulations. In thermal spike (TS) model, a set of coupled partial differential equations describes the heat conduction mechanism in the system^2,21,23^:

1 $${C}_{e}\frac{\partial {T}_{e}}{\partial t}=\nabla ({K}_{e}\nabla {T}_{e})+A(\bar{r},t)-g({T}_{e}-{T}_{l})$$

2 $${\rho }_{l}{C}_{l}\frac{\partial {T}_{l}}{\partial t}=\nabla ({K}_{l}\nabla {T}_{l})+g({T}_{e}-{T}_{l})$$

**Figure 1:**
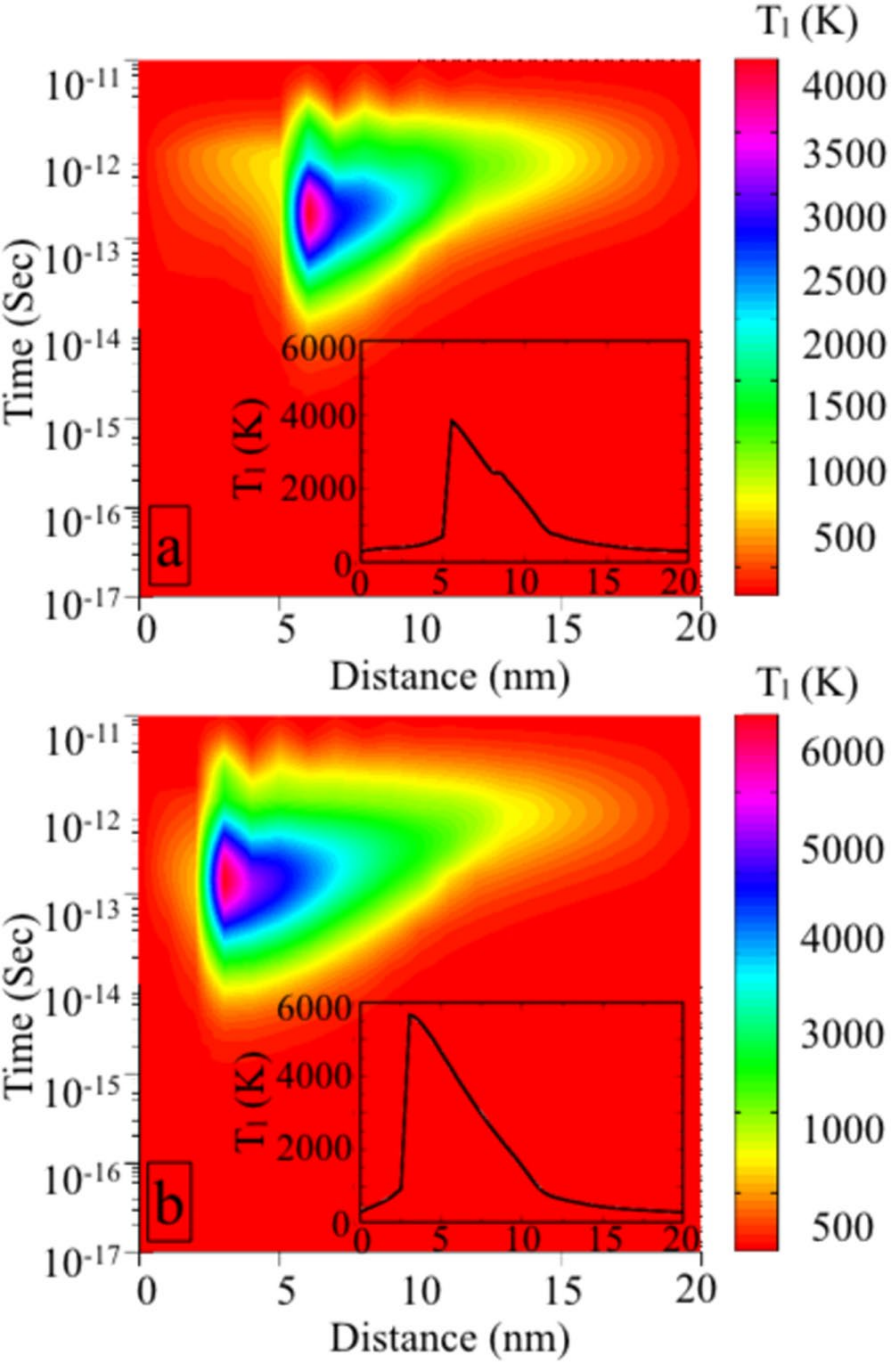
Lattice temperature (T_*l*_) rise as a function of distance from ion beam axis and time in (**a**) 5e13 and (**b**) 1e14 films as derived from thermal spike model calculations. Insets show the 2D projection of the T_*l*_ profiles varying with the distance for the respective films.

*C*_*e*_, *C*_*l*_, *K*_*e*_, *K*_*l*_ are the specific heats and thermal conductivities of electronic and lattice sub-systems respectively. *ρ*_*l*_ is the material density. *T*_*e*_ and *T*_*l*_ are the electronic and lattice temperatures. A($\bar{r}$, t) is the energy transferred to the electrons from heavy ion at a time t and at a distance r from the ion’s path. ${C}_{e}{|}_{{{\rm{SiO}}}_{2}}=1$ J/(cm^3^.K) and ${K}_{e}{|}_{{{\rm{SiO}}}_{2}}=2$ J/(cm.s.K); We have adapted the lattice specific heat and thermal conductivity values of Ni and SiO_2_ from the works of Wang *et al*.^37^ and Kumar *et al*.^2^. The details are given in refs^23,26^. Note that, the thermal relaxation time is much shorter than the mean time between successive ion impacts and therefore we have employed “single-ion impact” TS model. Although the quantitative determination of transient temperature rises for different fluences is beyond the limitations of the single ion-impact TS model, but the NP radius, which is not the same in different irradiated films, can be varied herein. The mean NP sizes are calculated to be 6 nm and 2.5 nm for 5e13 and 1e14 films from GISAXS and XTEM analysis^26^. Therefore, the lattice temperature rises obtained for different fluence irradiated films are nothing but the manifestation of the NP size modifications under SHI in our study. The lattice temperature of the Ni-SiO_2_ thinfilms rise to ≈4000 K and ≈6000 K within 10^−13^ to 10^−12^ sec for 5e13 and 1e14 films as shown in Fig. 1(a) and (b) respectively. Note that, consideration of simple 1D thermal spike model, as described in ref.^38^, leads to the temperature rise at the boundary of metal NPs and insulating matrix. This is mainly because we have considered scalar values of thermal conductivities in our modelling^39^. However, this does not affect the highest temperature values that we have used for our further analysis.

After getting the tentative highest T_*l*_ values for different irradiated NPs, the effect of SHI bombardment was further emphasized down to atomic level by *ab initio* molecular dynamic (MD) simulations. The Ni_38_ global minimum structure was then heated from room temperature to separately 1000, 4000 and 6000 K using MD simulations of 8 ps and then cooled down to 300 K via another MD simulation of 4 ps. For NVT MD simulation, we have used Nose thermostat throughout. Final structures at respective high (or low) temperatures were obtained on optimizing the MD structures. For all calculations, 0.01 meV was set as the convergence limit for achieving self-consistent total energy. To ensure high precision, a cut-off energy of 600 eV was chosen. For the optimization to ground-state geometry, atomic forces were reduced to less than 0.001 eV/Å by conjugated gradient (CG) minimization. Following this, on those preferred structures at respective temperatures, we have performed non-collinear magnetic calculations, which further tells us how the MA energy depends on the short-range spin-spin interaction and direction of magnetization.

## Results and Discussions

### Validation of chosen clusters and exchange-correlation functional

**Figure 2:**
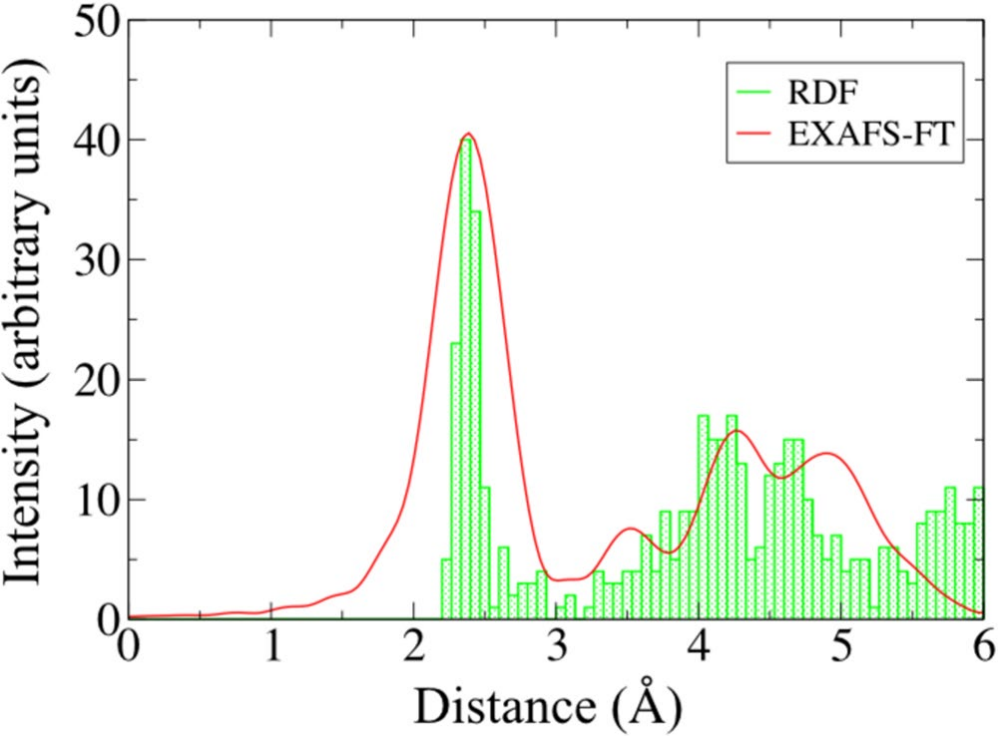
The Fourier transform of experimental EXAFS data for unirradiated film along with the RDF of corresponding MD structure.

Now, the calculated local structural and electronic structural properties, along with the atomic spin properties are quite sensitive to the choice of employed initial clusters. It is therefore of paramount importance to address whether the chosen clusters are sufficient in reproducing the experimental geometries (at various fluences) or not. Therefore, special attention is given in validating these aspects. In Fig. 2, the radial distribution function (RDF) as calculated from our *ab-initio* geometry at T = 1000 K is plotted alongside of the Fourier transform of the 45°-EXAFS data of the corresponding unirradiated film. The similarity in both the spectrum is direct indicative of the fact that the *ab-initio* MD structures adapted here are very well representing the experimental NPs as far as the local atomic environment is concerned. Note that, the RDF is calculated by estimating all possible bond-lengths of the clusters and thereby splitting them into equi-spaced bins. This takes into account the numbers of neighbouring atoms at particular distances i.e. how many atoms are there within the first co-ordination shell, how many are there in the second co-ordination shell etc.

**Figure 3:**
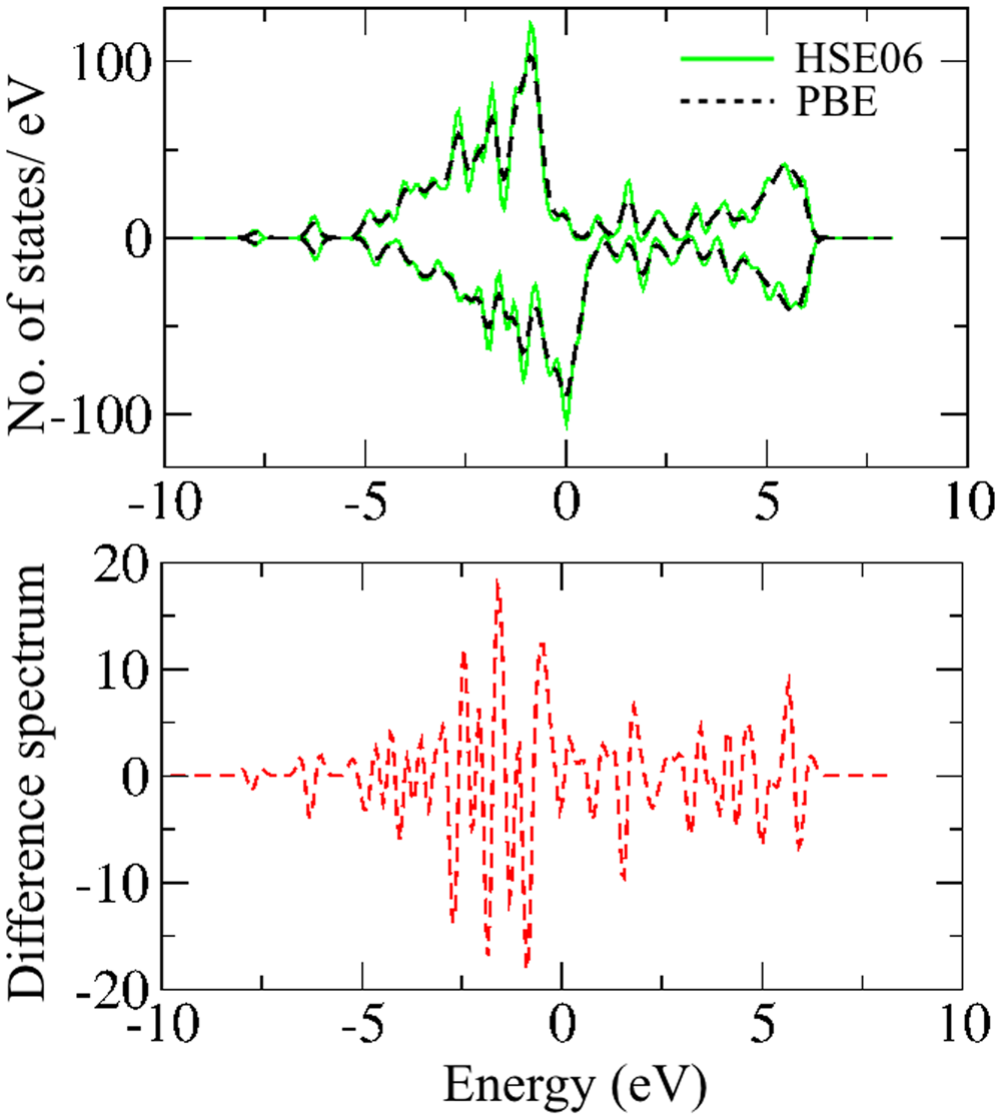
The DOS of T = 1000 K cluster calculated with PBE and HSE06. Bottom panel shows the corresponding difference spectrum.

In order to validate the DFT exchange-correlation (xc)-functional, we have next compared the performance of GGA xc-functional (viz. PBE) with respect to more advanced hybrid xc-functional (viz. HSE06). We have plotted spin-polarized density of states (DOS) of the T = 1000 K cluster (i.e. unirradiated film) using both PBE and HSE06 xc-functional as shown in Fig. 3. We clearly note from Fig. 3 (top-panel) that both the DOS match quite well with each other. The corresponding difference spectrum is (i.e. N_up_ (PBE) - N_up_ (HSE06)) shown in the bottom-panel. The resemblance of the spin polarized DOS of PBE with that of HSE06, thus, validates that all estimated magnetic properties are accurately determined at the level of PBE xc-functional.

### Observation of magnetic anisotropy and correlation with particle elongation

**Figure 4:**
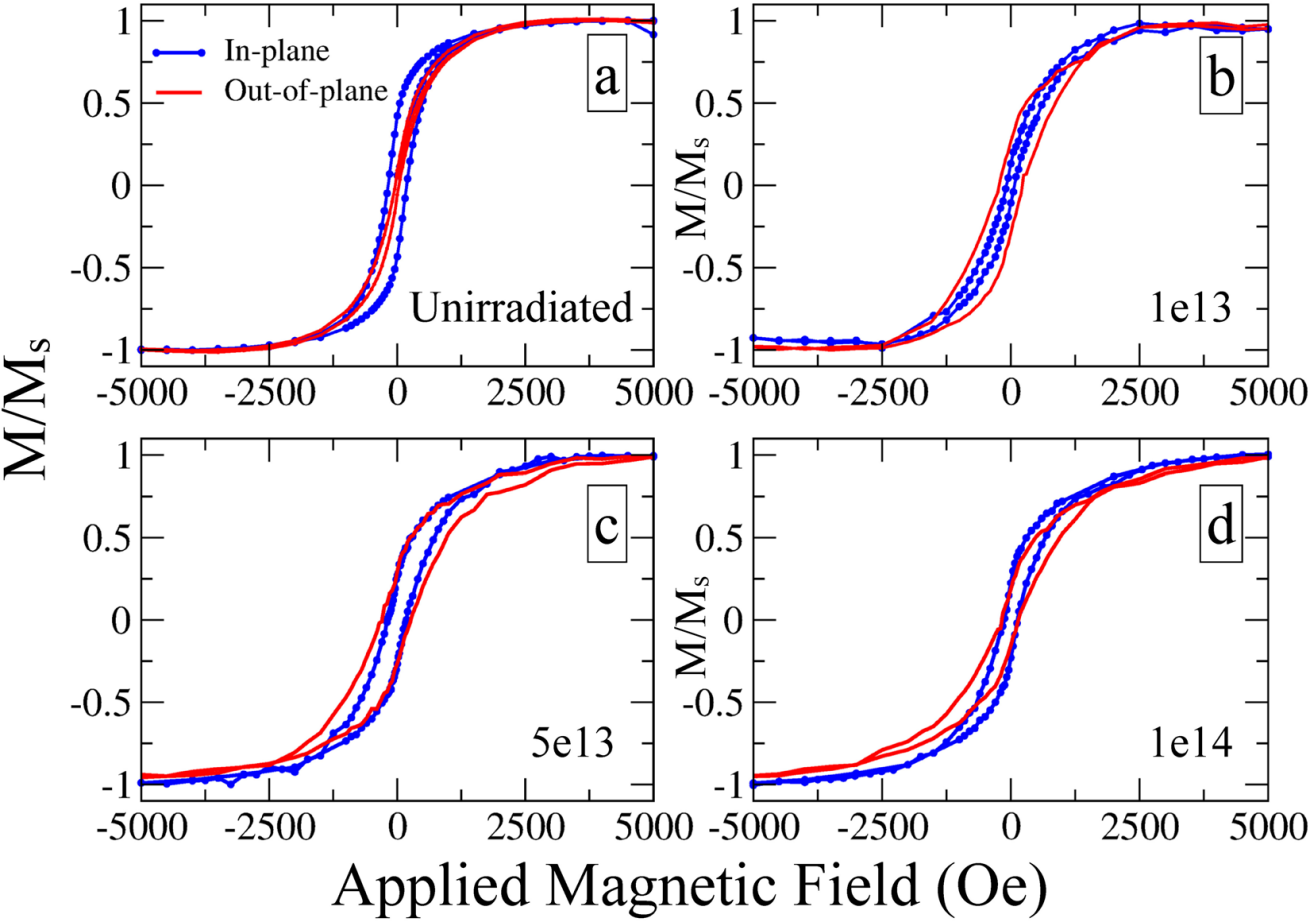
(**a**–**d**) In-plane and out-of-plane M-H plots at 5 K of unirradiated, 1e13, 5e13 and 1e14 films.

**Figure 5:**
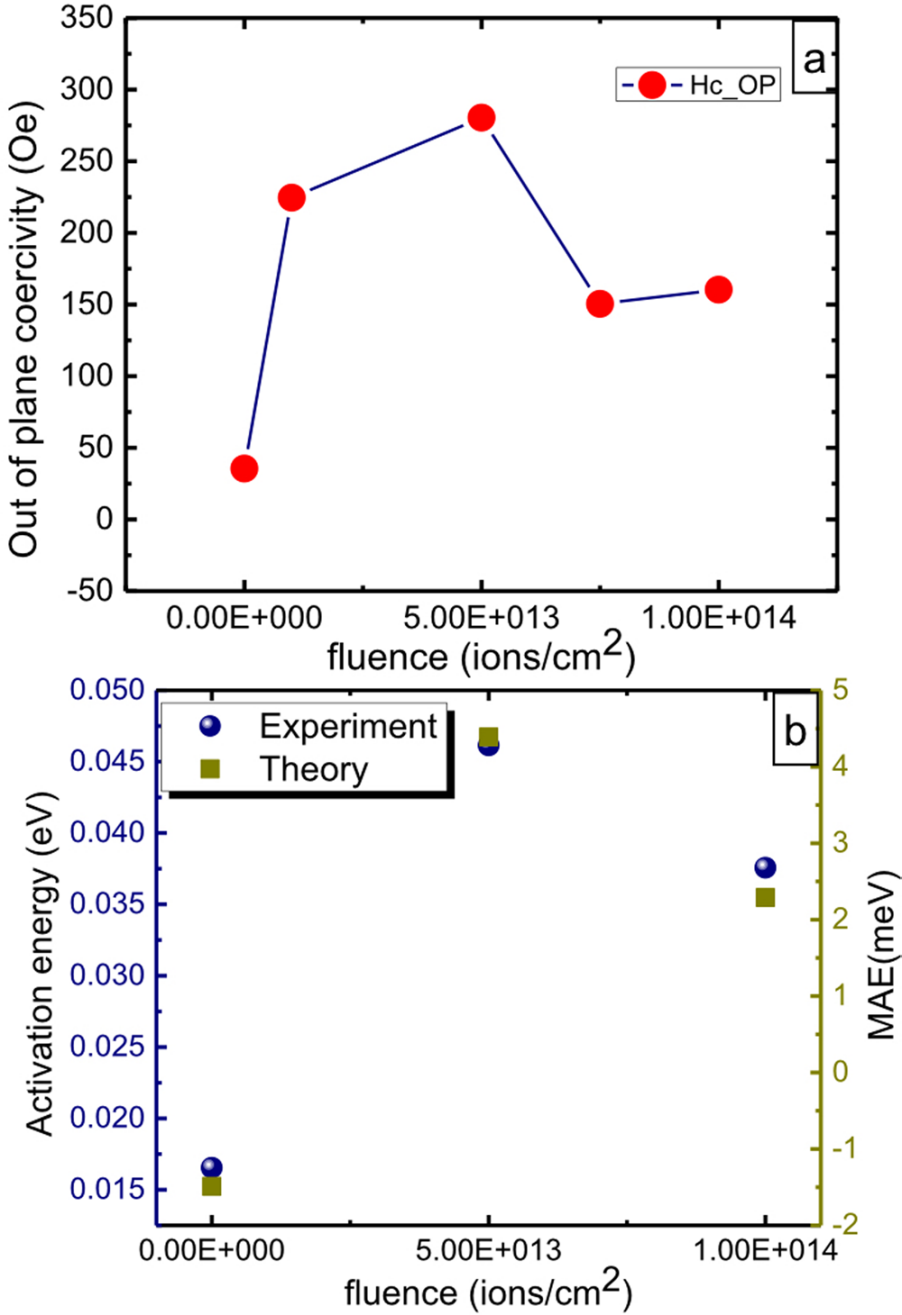
(**a**) out-of-plane coercivity vs irradiation fluence and (**b**) experimental and theoretical MAE values as function of irradiation fluences.

Figure 4(a–d) show the in-plane and out-of-plane (applied magnetic field parallel and perpendicular to film surface respectively) M-H plots for unirradiated and irradiated films. The magnetic anisotropy in terms of out-of-plane coercivity is maximum in the 5e13 film [Fig. 5a]. Further increase in SHI fluence reduces the coercivity. Note that, the saturation magnetization and coercivity in these composite films are ≈60–135 emu/cm^3^ and ≈50–300 Oe respectively, which are sufficiently larger values as compared to those observed before for this small fraction of Ni NPs inside any matrix^40^. The in-plane and out-of-plane coercivity (H_*c*_) and remanence ratio (M_*r*_/M_*s*_) of unirradiated and irradiated Ni-SiO_2_ films measured at 5 K are given in Table 1.

**Table 1:** In-plane and out-of-plane coercivity (H_*c*_ in Oe) and remanence ratio (M_*r*_/M_*s*_) of Ni-SiO_2_ films measured at 5K.

Sample Name	In-plane H_*c*_	Out-of-plane H_*c*_	In-plane M_*r*_/M _*s*_	Out-of-plane M_*r*_/M_*s*_
Unirradiated	36	30	0.42	0.09
e13	77	226	0.12	0.28
e13	177	283	0.20	0.28
e14	128	158	0.21	0.16

This magnetic behaviour in irradiated metal-insulator films is a manifestation of micro-structural and electronic structural properties. After initial reduction in Ni (111) peak height due to irradiation induced disordering^25^, not many changes are observed in the XRD pattern of other films irradiated at different fluences. Thus long-range ordering is not playing very crucial role in magnetic results obtained herein. The SHI stimulated elongation of embedded Ni NPs in 5e13 film and subsequent reduction in aspect ratio in 1e14 film becomes evident from our XTEM analysis [see Fig. 2 in ref.^26^]. Detailed GISAXS analysis has further confirmed that this trend is the same in entire film (figure not shown, detailed reported in our previous work^26^). Thus, shape modification is an obvious reason for induced magnetic anisotropy in 1e13 and 5e13 films. At the highest fluence (i.e. 1e14), Ni NPs loose their elongated shape, ending up in reduced out-of-plane coercivity. However, this observation is off-trend as MA has reportedly increased with increasing fluence in different metal NPs^10,41^. For in-depth understanding, therefore, we have first investigated the electronic structure of these electronically excited NPs by a combined experimental and first principles based calculations. The MA energy (MAE), calculated from both experimental ZFC-FC data and theoretical calculations (Fig. 5b) has the same trend i.e. it first increases at 5e13 and then again reduces at 1e14.

### Understanding from electronic structure: XPS and XANES

**Figure 6:**
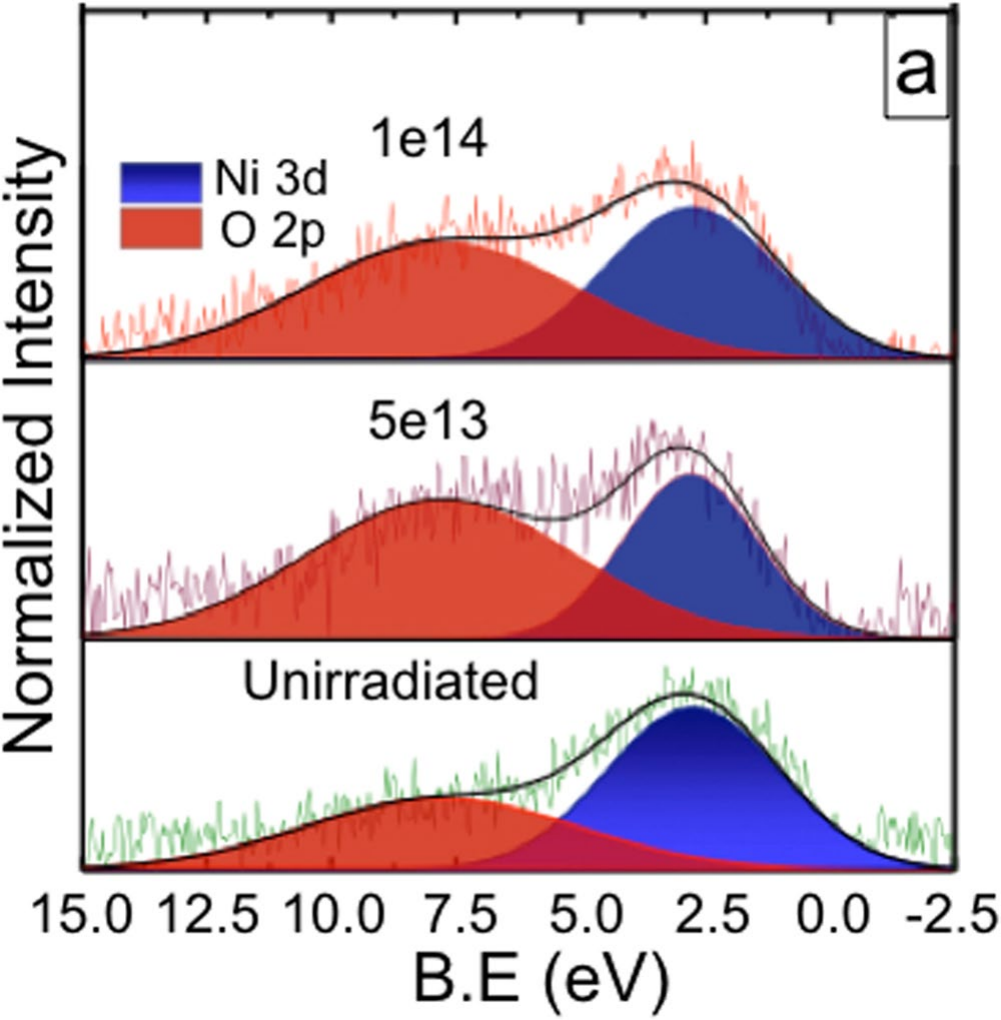
Valence band XPS of unirradiated, 5e13 and 1e14 films.

The magnetization of Ni, being an itinerant ferromagnet, is derived from the spin polarization of the itinerant d electrons. The sensitivity of these d electrons to local atomic environment makes the magnetic properties intertwined with both the electronic and atomic structures. High electron density of states near the Fermi level results in a spin polarized valence band and is reflected in the narrowest 3d electron band in the XPS valence band spectrum (Fig. 6) for the 5e13 film (keeping in mind the instrumental limitations). Higher fluence results in broadening of the same and affects the out-coming ferromagnetism. Note that, XPS is a surface sensitive technique and hence the O 2p signal arrises here as the surface gets partially oxidized post irradiation. A better insight of bulk-electronic structure [as detailed in ref.^26^] and its association with ferromagnetism is further scrutinized from pre-edge peak associated with transitions into mixed 3d-4p final states in XANES. The comparison with NiO and Ni reference spectra clearly signifies that the NPs sustain their metallic phase even after irradiation. Also, we did not find any signature of Ni-Si bonds from our model fitting of EXAFS data^26^. Pre-edge feature in XANES seems to decrease first and then again increases at higher fluence^26^. This suggests enhanced 3d-4p band overlaps (increased 3d bandwidth), resulting in diminished 3d electron correlations and a reduction in magnetic moment^13^.

For any itinerant ferromagnet, the Stoner criterion is given by I × D(E_*F*_) > 1. Here, D(E_*F*_) represents the density of states (DOS) at the Fermi level (E_*F*_), and I is the Stoner parameter (exchange integral), measuring the strength of exchange interaction. Since the value of the exchange interaction is very similar for all 3d transition metals (I ≈ 1 eV), D(E_*F*_) represents the most important parameter in the Stoner criterion. Thus the response of ferromagnetism to irradiation effect, in the first order approximation, is fully contained in it. In view of this, the observed 3d band-broadening from both XPS and XANES at higher fluence is due to the decreased 3d DOS at the Fermi level, which reduces D(E_*F*_) term in Stoner equation. This has immediate effect to weaken the magnetic response as we go on increasing the SHI fluence beyond 5e13. We also note from the DOS of different clusters [see SI] that, the T = 1000 K structure still holds the similarity with bulk fcc Ni DOS, which is obvious as the unirradiated film contains fcc Ni NPs. However, the nature changes at T = 4000 K. We observe broadening of band near E_*F*_: indicative of reduced Ni-Ni ordering in the irradiated films. Also, note that the majority spin states have zero occupancy for both the clusters at E_*F*_, whereas the minority states are occupied. Moreover, the change in DOS is pronounced for the minority orbitals. We can therefore say that the anisotropy and changes in magnetization is overruled by the minority spins.

### Influence of local atomic structure: angle dependent EXAFS and DFT

**Figure 7:**
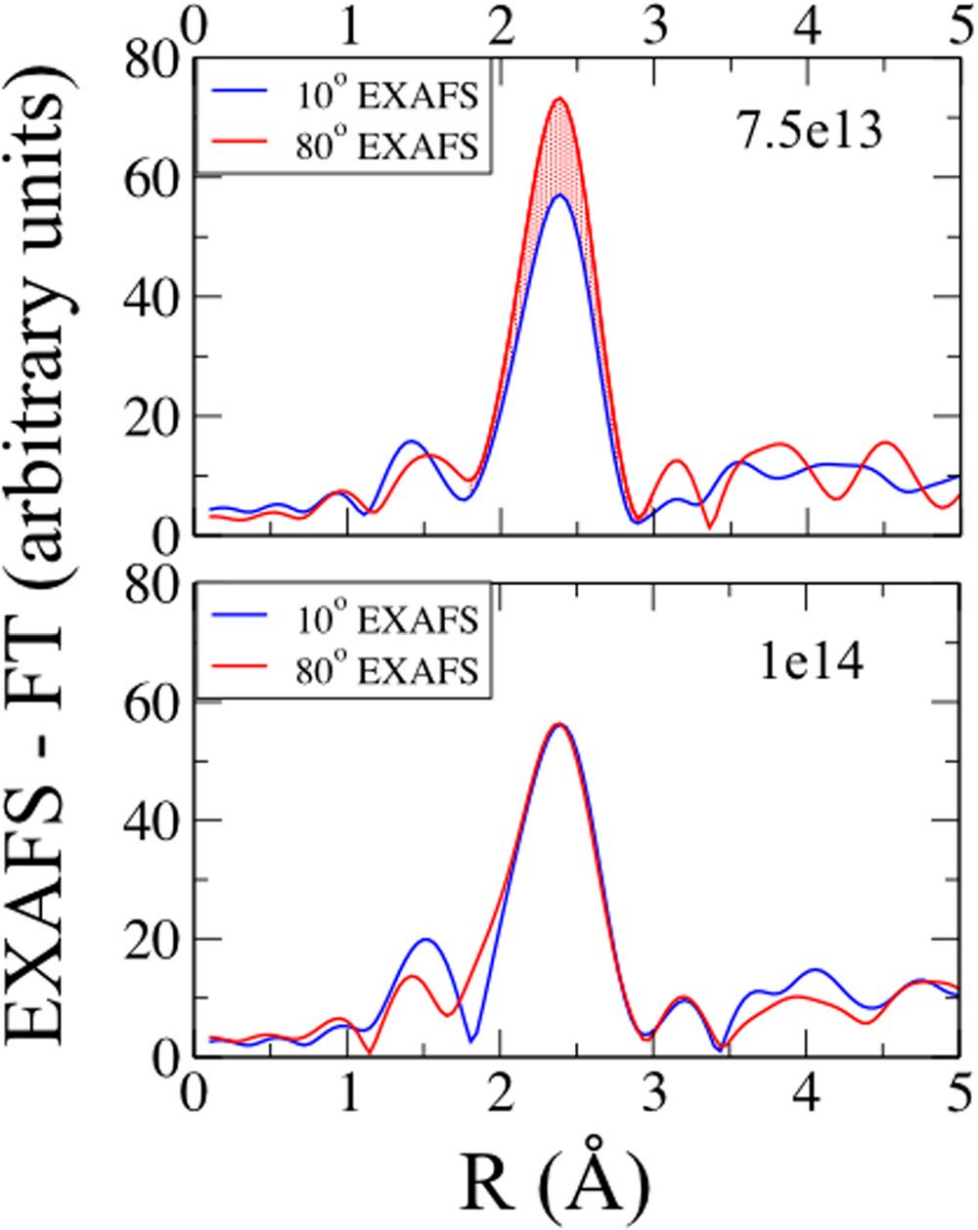
Angle dependent EXAFS data for unirradiated and irradiated films showing the structural anisotropy in 7.5e13 film and its reduction beyond.

Coming to the local atomic environment now, we see in Fig. 7 the Fourier transforms of the EXAFS data collected for different incident angles of the incoming X-rays for 7.5e13 and 1e14 films. The local structural anisotropy at intermediate fluence can easily be identified from the directional local structural probe for 7.5e13 film, where the 10° and 80° EXAFS are not identical. Note that, the atomic disorder coupled with structural anisotropy has resulted in improved MA in Ni NPs at intermediate fluences. However, beyond 7.5e13 the structural anisotropy is not persistent and the two different angled EXAFS get merged in 1e14 film. The overlapping of the EXAFS at both 10° and 80° in case of 1e14 film is similar to unirradiated film. This demonstrates the homogeneity in local atomic environment. Also, the decrease in EXAFS signal amplitude is a direct consequence of enhanced structural disorder at the highest fluence. Therefore, a direct correlation between the local structural anisotropy and MA is present.

**Figure 8:**
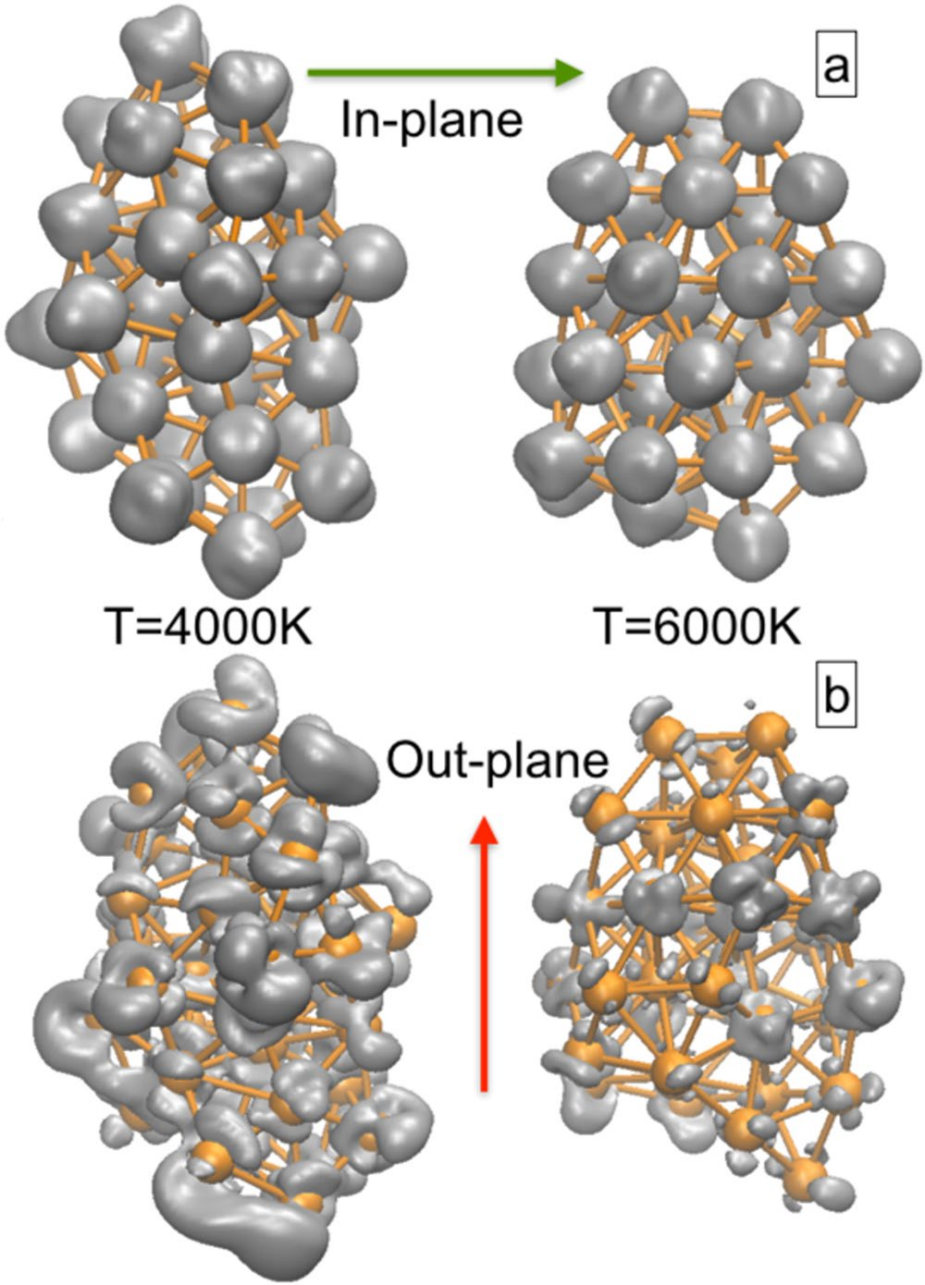
Spin isosurface plots (grey) for T = 4000 K and T = 6000 K structures when magnetic field is applied along (**a**) minor axis and (**b**) major axis. Orange spheres represent Ni atoms.

This observed experimental local structural anisotropy is further realized from our first-principles calculations by addressing the role of spin-spin interactions in controlling the atomic arrangements. We have demonstrated in our earlier work that the structural anisotropy in the irradiated Ni NPs can only be realized if we take into account the atomic spin-spin interactions^26^. Here we have plotted the spin density isosurfaces of different MD structures from our non-collinear magnetic calculations, where the applied field is given (a) along minor dimension (i.e. in plane) and then (b) along major dimension (i.e. out of plane). In Fig. 8 we can clearly see that the changes in the local atomic-arrangement in different MD structures (at T = 4000 K and T = 6000 K) have also brought changes in the spin density. We see that directional magnetic anisotropy is prominent in T = 4000 K structure due to the variation in spin alignments when the direction of applied magnetic field is altered. At T = 6000 K, not only the structure gets modified, but also it has lost it’s spin alignment when the field is applied along the major dimension. This explains why at higher fluence (i.e higher temperature) we encounter reduced MA. Therefore, the local structural inhomogeneity has invited the directional spin inhomogeneity in the ferromagnetic system, which in turn modifies the MA. The change in aspect ratio and the differences in density of states of the respective MD clusters can be found in SI.

## Conclusion

In summary, elongation and consequent magnetic axial anisotropy of embedded Ni NPs can be tuned by optimizing the SHI fluences. The maximum MA at the intermediate fluences, is not only the outcome of dimensional aspect ratio but is also strongly inter-winded with the local structural anisotropy, which indeed is manifested by atomic spin-spin interaction. At very high fluence, Ni NPs loose their elongated shape and the MA gets reduced. The randomisation of spin alignment plays an instrumental role in controlling the local structural anisotropy and hence the MA. Therefore, the understanding of structure mediated and tuneable MA may lead to design of ferromagnetic artificial patterns inside insulator media by SHI that offers application in high-density perpendicular storage.

## Electronic supplementary material

Supplementary information
