# Supplementary material for: Evidence of local structural influence on the shape driven magnetic anisotropy in electronically excited Ni nanoparticles embedded in SiO2 matrix
Source: Sci Rep. 2018 Jan 18;8:1040. doi: 10.1038/s41598-017-18731-x (PMC5773491; doi:10.1038/s41598-017-18731-x)
Supplement: Supplementary file 1 — Supplementary information [file 41598_2017_18731_MOESM1_ESM.pdf]

Evidence of local structural influence on the shape driven magnetic anisotropy in electronically excited Ni nanoparticles embedded in SiO_2 matrix

Debalaya Sarker[1*], Saswata Bhattacharya[1], H. Kumar[1+], Pankaj Srivastava[1], and Santanu Ghosh[1*]

[1]Department of Physics, Indian Institute of Technology Delhi, Hauz Khas 110016, New Delhi, India

[*]debalaya.sarker@physics.iitd.ac.in (DS), santanu1@physics.iitd.ac.in (SG)

[+]Present address: Department of Applied Physics, Amity University U.P. , Sector 125 Noida, 201301, India

Supporting information

Aspect Ratio of clusters:

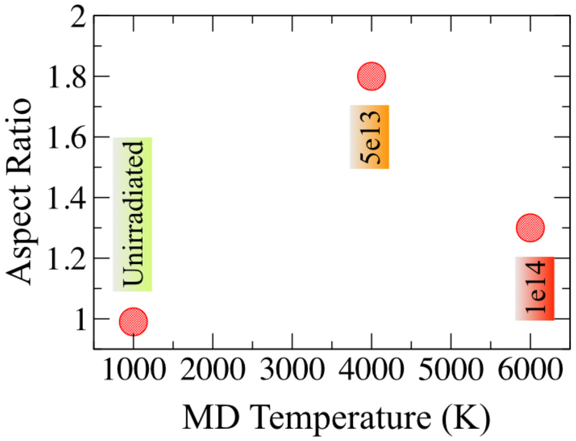

Figure S1: Aspect ratio of different MD clusters as a function of MD temperature; corresponding SHI fluences are shown in boxes.

The aspect ratio changes from 0.99 → 1.8 → 1.3 as one goes from T=1000K → T=4000K → T=6000K cluster respectively. We do not find any significant change in the

1st shell Ni-Ni bond-length among different clusters, which is in agreement with our experimental 45°-EXAFS analysis.

Density of states (DOS) of different MD-clusters:

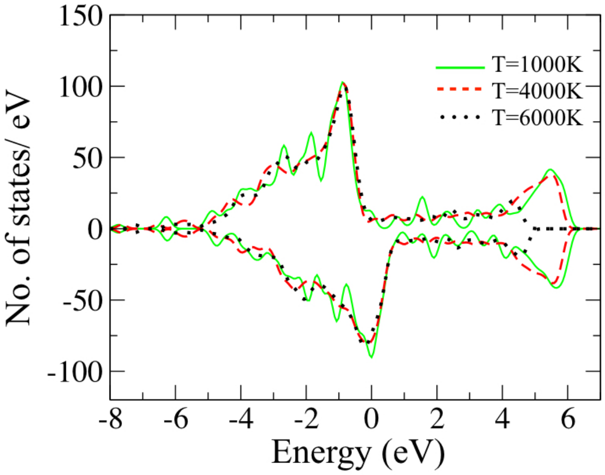

Figure S2: Density of states of different MD clusters.

We note that, T=1000K structure's DOS still holds the similarity with bulk fcc Ni DOS, which is obvious as the unirradiated film contains fcc Ni NPs. However, the nature changes at T=4000K. We observe broadening of band near Ef (= 0 eV): indicative of reduced Ni-Ni ordering in the irradiated films. We note that, T=1000K structure's DOS still holds the similarity with bulk fcc Ni DOS, which is obvious as the unirradiated film contains fcc Ni NPs. However, the nature changes at T=4000K. We observe broadening of band near Ef (= 0 eV): indicative of reduced Ni-Ni ordering in the irradiated films.
